# Molecular and Biochemical Characterization of a Type II Thioesterase From the Zoonotic Protozoan Parasite *Cryptosporidium parvum*

**DOI:** 10.3389/fcimb.2019.00199

**Published:** 2019-06-07

**Authors:** Fengguang Guo, Haili Zhang, Rana Eltahan, Guan Zhu

**Affiliations:** Department of Veterinary Pathobiology, College of Veterinary Medicine & Biomedical Sciences, Texas A&M University, College Station, TX, United States

**Keywords:** apicomplexan, *Cryptosporidium parvum*, fatty acid synthase (FAS), polyketide synthase (PKS), α/β-hydrolase, type II thioesterase (TEII)

## Abstract

*Cryptosporidium parvum* is a globally important zoonotic parasite capable of causing severe to deadly diarrhea in humans and animals. Its small genome (~9.1 Mb) encodes not only a highly streamlined metabolism, but also a 25-kb, 3-module fatty acid synthase (CpFAS1) and a 40-kb, 7-module polyketide synthase (CpPKS1). The two megasynthases contain a C-terminal reductase domain to release the final products with predicted chain lengths of ≥C22 for CpFAS1 or C28 to C38 for CpPKS1.The parasite genome also encodes a discrete thioesterase ortholog, suggesting its role to be an alternative tool in releasing the final products from CpFAS1 and/or CpPKS1, or as an editor to remove non-reactive residues or aberrant intermediates, or to control starter units as seen in other parasites. In this study, we have confirmed that this *C. parvum* thioesterase is a type II thioesterase (thus named as CpTEII). CpTEII contains motifs and a catalytic triad characteristic to the type II thioesterase family. CpTEII is expressed during the entire parasite life cycle stages with the highest levels of expression in the later developmental stages. CpTEII showed the highest hydrolytic activity toward C10:0 decanoyl-CoA, so we speculated that CpTEII may mainly act as an editor to remove non-reactive residues and/or aberrant medium acyl chain from CpFAS1 and/or CpPKS1. However, we cannot rule out the possibility that CpTEII may also participate in the release of final products from CpFAS1 because of its moderate activity on C20:0, C:22:0 and C24:0 acyl-CoA thioesters (i.e., ~20–30% activity vs. decanoyl-CoA).

## Introduction

*Cryptosporidium parvum* is a protozoan parasite, which belongs to the Phylum Apicomplexa and is a causative agent of cryptosporidiosis in humans and various animals (Tzipori and Widmer, 2008; Ryan et al., 2014). *Cryptosporidium* may cause severe to deadly opportunistic infections in immunocompromised patients, and is listed as a category B priority pathogen in the NIH/CDC biodefense program (Chen et al., 2002; Rotz et al., 2002). Moreover, cryptosporidiosis is associated with high morbidity and mortality rate worldwide, and is one of top four pathogens causing moderate to severe diarrhea in infants under the age of two in developing countries (Kotloff et al., 2013; Checkley et al., 2015).

The zoonotic *C. parvum* possesses relatively small genome (~9.1 Mb) that encodes a highly streamlined metabolism and lacks enzymes for *de novo* synthesis of amino acids, nucleotides, or fatty acids (Abrahamsen et al., 2004; Xu et al., 2004). On the other hand, this parasite has two megasynthases: a 921 kDa type I fatty acid synthase (CpFAS1) and a 1,516 kDa type I polyketide synthase (CpPKS1) (Zhu et al., 2002, 2004, 2010). These two megasynthases are unable to synthesize fatty acyl or polyketide chains *de novo*, but likely responsible for synthesizing very long-chain fatty acyl or polyketide alcohols using long-chain fatty acids as the starter units, because both CpFAS1 and CpPKS1 have been confirmed to have substrate preference toward long-chain fatty acids. Also, terminating domain of CpFAS1 prefers to hydrolyze very long-chain fatty acids (Fritzler and Zhu, 2007; Zhu et al., 2010).

Despite of lacking type II FAS or PKS, the *Cryptosporidium* genomes encode a discrete thioesterase ortholog with conserved motifs characteristic to the type II TE (TEII) in the α/β-hydrolase superfamily. A large number of prokaryotic and eukaryotic TEIIs have been reported to play diverse roles, ranging from the removal of non-reactive residues or aberrant intermediates, control of starter units, providing key intermediates, to the release of products (Kotowska and Pawlik, 2014). Up to date, no TEII enzymes have been reported and characterized in any protozoa.

In the present study, we report the characterization of the molecular and biochemical features of a TEII from *C. parvum* (CpTEII) for the first time in a protozoan. We have confirmed CpTEII's hydrolysis activity on fatty acyl-CoA thioesters. CpTEII displayed the highest activity on the C10:0 decanoyl-CoA, suggesting that CpTEII may mainly play an editing role by removing aberrant or non-reactive medium chains from CpFAS1 and/or CpPKS1 assembly in *C. parvum*.

## Materials and Methods

### Molecular Sequence Analysis

A discrete thioesterase (TE) gene was identified from the *C. parvum* genome (GenBank accession number: XM_628403) that was annotated as “thioesterase of the a/b hydrolase superfamily, possible bacterial origin” by the genome sequencing project (gene ID: cgd7_2320 at http://www.CryptoDB.org) (Abrahamsen et al., 2004). To validate the annotation and predict the function, its amino acid sequence (XP_628405) was used as a query to search orthologs against the non-redundant protein sequence databases at NCBI using BLASTP algorithm (https://blast.ncbi.nlm.nih.gov/). Top hits with query cover ≥50% and expect value ≤ 1e-5 were retrieved from the databases for conducting multiple sequence alignment using T-coffee algorithm implanted in the MacVector program (version 15.0 or higher). The nearly identical sequences were removed from the dataset, and the gap and insertion regions in the alignment file were manually removed. The final dataset containing 32 sequences were aligned again using T-Coffee algorithm to identify conserved motifs. The conserved domains in CpTEII was also identified by searching the NCBI conserved domains databases (https://www.ncbi.nlm.nih.gov/Structure/cdd/wrpsb.cgi).

### Cloning of *CpTEII* Gene and Expression of Recombinant CpTEII Protein

Because *CpTEII* gene contained no intron, its entire open reading frame (ORF) was amplified from the *C. parvum* genome DNA using Pfu DNA polymerase (Agilent, Santa Clara, CA) and primers CpTEII_F1_BamHI (5′ GTGGATCCATGTCAAAATCAGATTTC 3′) and CpTEII-R1_HindIII (5′ CCAAGCTTAATAGTATTCTAGGTCTAATA 3′) (linker sequences are underlined). The PCR products were digested with *Bam*HI and *Hind*III and ligated into the expression vector pMAL-c2e-TEV that contained an N-terminal maltose-binding protein (MBP) tag and a C-terminal His-tag (Guo and Zhu, 2012). The plasmid was transformed into One Shot TOP10 competent *Escherichia coli* (Invitrogen, Carlsbad, CA), followed by an overnight growth of transformed bacteria in a lysogeny broth (LB) agar plate containing 100 μg/mL ampicillin. Bacterial colonies containing *CpTEII* inserts were identified by PCR directly using colonies as templates and primers flanking the vector and insert. Plasmids were isolated from positive colonies and sequenced to identify those containing inserts with correct orientation and sequence. The construct was named as pMAL-c2e-TEV-CpTEII.

The expression of recombinant MBP-fused CpTEII protein was performed in the Rosetta 2 strain of *Escherichia coli* cells that contains an extra set of tRNA genes for 7 rare codons to enhance the expression of eukaryotic proteins (EMD Biosciences, Madison, WI). A colony of Rosetta 2 transformants was inoculated in 50 mL of LB medium containing ampicillin (50 μg/mL), chloramphenicol (34 μg/mL), and glucose (2 mg/mL). After incubation at 37°C with shaking at 200 rpm overnight, bacterial suspension was diluted by 1:20 with 1.0 L fresh LB medium containing antibiotics and glucose, and incubated at 37°C for ~2 h or until the OD_600_ reached to ~0.5. At this time point, protein expression was induced by adding isopropyl-1-thio-β-D galactopyranoside (IPTG) at a final concentration of 0.3 mM, followed by incubation at 16°C for ~16 h with shaking. The cells were harvested by centrifugation and stored in −20°C overnight or until use. The frozen cells were resuspended in 20 mL ice-cold column buffer (20 mM Tris-HCl, pH 7.4, 200 mM NaCl) containing a protease inhibitor cocktail for bacteria (Sigma-Aldrich, St. Louis, MO), and lysed by sonication (Guo and Zhu, 2012). Bacterial lysates were centrifuged at 10,000 × *g* at 4°C for 30 min to collect supernatants. The recombinant MBP-CpTEII protein was purified from the supernatants using an amylose resin-based affinity chromatography following the manufacturer's protocol (New England Biolabs, Ipswich, MA). In some experiments, purified MBP-CpTEII was incubated with tobacco etch virus protease (TEV) (TEV: MBP-CpTEII = 1:50) at 4°C overnight to cleave the MBP tag from the fusion protein and the cleaved CpTEII were further purified using Ni-NTA agarose (Qiagen Inc. MD) following the product manual.

### Biochemical Assays

The hydrolysis activity of CpTEII was measured by taking advantage that most TEIIs were capable of using acyl-CoA as substrates in addition to the acyl-ACP (acyl-carrier protein). The release of CoA of fatty acyl-CoA after hydrolysis was spectrophotometrically monitored using 5,5′-Dithio-bis-(2nitorbenzoate) (DTNB; Ellman's reagent), in which free CoA in reduced form (CoA-SH) reacted with DTNB to form 5-thionitrobenzoic acids that could be measured at 412 nm (Kotowska et al., 2009; Guo et al., 2014). A typical assay was performed in 200 μL of HEPES buffer (0.1 M, pH 7.4) containing 50 mM of KCl, 50 μM of a specified fatty acyl-CoA, 50 μM of DTNB and 10 μg of MBP-CpTEII or MBP-tag only as a negative control for background subtraction. The reaction was started with the additions of acyl-CoA (50 μM final concentration) into the premixed reaction cocktails, and the absorbance was recorded at 412 nm every min for 30 min. The substrate preference of CpTEII was evaluated individually using a set of fatty acyl-CoAs (50 μM) with varied chain lengths (i.e., from C4:0 to C24:0). For substrate preference assay, bovine serum albumin (BSA) was included in reaction solutions (molar ratio between BSA and acyl-CoA = 1:4.5). Detailed enzyme kinetics was measured using C10:0 decanoyl-CoA that was the most preferred substrate by CpTEII. For testing the effect of pH on CpTEII activity, citric acid–Na_2_HPO_4_ (pH 5.5–7.0) and HEPES (pH 7.0–9.0) buffer systems were used. All acyl-CoA thioesters used in this study were purchased from Avanti Polar Lipids (Alabaster, AL) and other chemicals were purchased from Sigma-Aldrich (St. Louis, MO). The data were analyzed using Prism (version 5.0f or higher; GraphPad Software, La Jolla, CA), and the Tukey's multiple comparisons test was used to analyze the effect of pH on the CpTEII activity.

### Analysis of *CpTEII* Gene Expression by qRT-PCR

Total RNA was isolated from *C. parvum* oocysts, sporozoites, and various intracellular stages of the parasite developed in HCT-8 cells using RNeasy mini kit (Qiagen Inc., Valencia, CA, US). The relative expression levels of *CpTEII* were evaluated by quantitative real-time RT-PCR (qRT-PCR) using One-Step RT-PCR QuantiTect SYBR Green RT-PCR Kit (Qiagen Inc., Valencia, CA) and primers CpTEII-566F (5′ TCC CGG AAT ATG ACC GTC CAT GGA 3′) and CpTEII-645R (5′ TCC CCA CTC TCT GCA CTC TTG T 3′) for *CpTEII* and Cp18S-1011F (5′ TTG TTC CTT ACT CCT TCA GCA C 3′) and Cp18S-1185R (5′ TCC TTC CTA TGT CTG GAC CTG 3′) for the parasite 18S rRNA (*Cp18S*). The qRT-PCR was performed as previously described (Zhang and Zhu, 2015). Briefly, each 20 μL of reaction mixture contained 500 nM of each primer, 0.25 μL of RT master mix, 1X QuantiTect SYBR-Green and 1.0 ng of total RNA from oocysts or sporozoites, or 15.0 ng of total RNA from infected cells. The mixtures were incubated at 50°C for 30 min to synthesize cDNA, and then heated at 95°C for 15 min to inactivate the reverse transcriptase, followed by 40 thermal cycles of PCR amplification (95°C for 20 s, 58°C for 30 s, and 72°C for 30 s) on an CFX Connect Real-Time PCR Detection System (Bio-Rad Labs, Hercules, CA, USA). At least two technical replicated qRT-PCR reactions were performed for each sample.

The relative levels of *CpTEII* transcripts were calculated by an empirical $2^{{-\Delta \Delta C}_{T}}$ formula as described below. Briefly, the levels of *CpTEII* transcripts were first normalized with those of *Cp18S* transcripts by calculating the Δ*C*_T_ values between *CpTEII* and *Cp18S* transcripts in individual samples:

(1) $$\begin{array}{l} \Delta C_{\text{T}}=C_{\text{T}\left[\text{CpTEII}\right]}-C_{\text{T}\left[\text{Cp}18\text{S}\right]} \end{array}$$

Then the relative levels of *CpTEII* transcripts were determined using that in the *C. parvum* oocysts as the baseline based on the ΔΔC_T_ values between all developmental stages and oocysts:

(2) $$\begin{array}{l} \Delta \Delta C_{\text{T}}=\Delta C_{\text{T}\left[\text{sample}\right]}-\Delta C_{\text{T}\left[\text{oocysts}\right]} \end{array}$$

(3) $$\begin{array}{l} Relative\;level\;of\;CpTEII\;transcripts=2^{{-\Delta \Delta C}_{T}} \end{array}$$

### Detection of CpTEII Protein in the Parasite

The methods in the production of polyclonal antibodies, western blot analysis, and immunofluorescence microscopy of CpTEII protein are briefly described as follows. More details on the general procedures can be found in previous reports for detecting other proteins in *C. parvum* (e.g., Zhang et al., 2012, 2015; Guo et al., 2016).

#### Production and Purification of Anti-CpTEII Polyclonal Antibodies

Polyclonal antibodies against CpTEII were produced in rabbits by Alpha Diagnostic International (San Antonio, TX). The purified recombinant CpTEII was used as an antigen to immunize two specific pathogen-free (SPF) rabbits for antibody production using a standard 63-day protocol, which included 5 injections at multiple sites in 14-day intervals. Pre-immune bloods were collected prior to immunization, and antisera were collected twice at 7 wk and 9 wk after the immunization.

The anti-CpTEII polyclonal antibodies were purified using an antigen-based affinity purification protocol as previously described but with minors modifications (Kurien, 2009; Guo et al., 2016). Briefly, the recombinant CpTEII antigen was separated by a 10% SDS-PAGE gel and transferred onto nitrocellulose membranes. The blots were stained with Ponceau Red, and the strip containing the recombinant protein was cut out for subsequent antibody purification. The strip was blocked with 5% BSA in TBST (Tris-buffered saline containing 0.02% Tween 20) for 1 h, and then incubated with antisera overnight at 4°C on an orbital shaker. The strip was washed three times with TBST (10 min each), and antibodies that bound to the antigen on the membrane were eluted in 0.2 M glycine (pH 2.2) by vigorous shake on an orbital shaker for 2 min, followed by neutralization with 1.0 M Tris-HCl buffer (pH 8.0).

#### Western Blot Analysis of CpTEII

The parasite whole protein was extracted from oocysts and free sporozoites. The free sporozoites were prepared by incubating *C. parvum* oocysts in PBS containing 0.5% taurodeoxycholic acid (TDC) and 0.25% trypsin for 45 min at 37°C. Released sporozoites were collected and washed with culture medium containing 10% fetal bovine serum (FBS) to neutralize trypsin. The oocysts or freshly prepared sporozoites were lysed in 100 μL of radioimmunoprecipitation assay (RIPA) buffer (Pierce Biotechnology, Rockford, IL) containing a protease inhibitor cocktail for eukaryotes (Sigma-Aldrich, St. Louis, MO). The lysates (equivalent to ~4 × 10^7^ sporozoites/lane) were fractioned on a 4–20% Mini-PROTEAN TGX Stain-Free Protein Gel (Bio-Rad, Hercules, CA) and imaged using ChemiDoc XRS+ system prior to the transfer of proteins onto nitrocellulose membranes.

The blots were blocked with 5.0% fat-free milk in TBST (50 mM Tris-HCl, pH 7.5; 150 mM NaCl and 0.05% Tween-20) for 1 h, incubated with affinity-purified anti-CpTEII polyclonal antibody (1:1,000 dilution in 5% fat-free milk-TBST) for 1 h, and then incubated with horseradish peroxidase-conjugated goat anti-rabbit IgG secondary antibody (1:20,000 dilution in 5% fat-free milk-TBST) for 1 h. Three washes with TBST (15 min each wash) were applied after each incubation step. The blots were developed using a SuperSignal™ West Femto maximum sensitivity substrate according to the manufacturer's protocol (ThermoFisher Scientific) and imaged using ChemiDoc XRS+ system (Bio-Rad). For antibody specificity test, the same western blotting procedure was performed except that the anti-CpTEII antibodies were presoaked with recombinant CpTEII protein prior to incubation with the blot. In brief, the diluted anti-CpTEII antibodies were incubated with the recombinant CpTEII proteins that were immobilized on nitrocellulose membrane, and then after 1-h incubation, the pretreated anti-CpTEII antibodies were used in the following western blotting.

### Indirect Immunofluorescence Microscopic Detection of CpTEII

The sporozoites of *C. parvum* were prepared by an *in vitro* excystation procedure as described above. Intracellular stage parasites in HCT-8 cells were prepared by infecting HCT-8 cell monolayers grown on glass coverslips with *C. parvum* oocysts for 3 h before washing out unexcysted oocyst and debris, and then adding fresh culture medium and let the parasite grow for total 6, 12, 24, 36, 48, or 72 h before fixing the samples. All samples were fixed in 4% formaldehyde for 30 min at room temperature. Fixed cells were washed with PBS and permeabilized with 0.1% Triton X-100 in PBS for 5 min. Samples were then blocked with 5% FBS in PBS for 60 min, followed by incubation with affinity-purified anti-CpTEII antibodies and previously described chicken anti-CpPKS IgY(1:100 dilution in 5% FBS-PBS) (Zhu et al., 2002) for 60 min, followed by incubation with 1:1,500 diluted donkey anti-chicken IgY-CF488A and donkey anti-Rabbit-CF568 secondary antibodies (Biotium Inc. Fremont, CA) for 60 min. After each incubation, samples were then washed three times with 5% FBS-PBS for 10 min. After the last wash, samples were mounted onto glass slides with a Prolong Gold Antifade reagent containing 4',6-diamidino-2-phenylindole (DAPI) for counter-staining of nuclei (Molecular Probes/Invitrogen, Grand Island, NY). Cells were then examined with an Olympus BX51 research microscope equipped with appropriate filter sets. Images were captured with a Retiga SRV CCD Digital Camera (QImaging, Surrey, BC) and processed uniformly with Adobe Photoshop version CS4 or higher.

## Results

### CpTEII Protein Contains Motifs and Active Sites Characteristic to Type II Thioesterase

**Figure 1:**
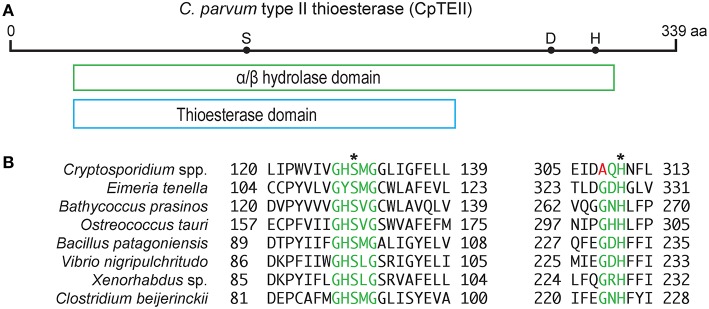
CpTEII contains motifs and catalytic triad characteristic to type II thioesterase. **(A)** CpTEII (339 aa) is largely composed of a conserved domain belonging to the α/β hydrolase superfamily (cl21494), which includes GrsT (Surfactin synthase thioesterase subunit; COG3208) and thioesterase (pfam00975) domains. Dots represent the three active site residues S^128^, D^278^, and H^309^. **(B)** Alignments at the two motifs conserved in TEIIs from various organisms. Motif GxSxG (green) is conserved among most species, while motif GxH conserved in most prokaryotic TEIIs is less conserved in *Cryptosporidium* species by the replacement of Gly residue with Ala. We hence revised the motif sequence to (G/A)xH. Asterisks (*) indicate two of the three catalytic sites located within the two motifs. GenBank accession numbers for the sequences: XP_628405, *Cryptosporidium parvum*; XP_013228501, *Eimeria tenella*; XP_007508988, *Bathycoccus prasinos*; XP_003074702, *Ostreococcus tauri*; WP_078393735, *Bacillus patagoniensis*; CCO57165, *Vibrio nigripulchritudo*; WP_099111618, *Xenorhabdus sp*.; WP_017210296, *Clostridium beijerinckii*.

The *CpTEII* gene encodes 339 amino acids with predicted molecular weight of 39 kDa, which is slightly larger than a typical type II thioesterase (25–29 kDa) (Kotowska and Pawlik, 2014). CpTEII were highly homologous to the orthologs from other *Cryptosporidium* species, but much less homologous to the TEII enzymes from distant prokaryotes or eukaryotes in BLASTP searches of the non-redundant NCBI protein databases (Figure S1). The majority of the CpTEII sequence is contained by an α/β hydrolase fold and a thioesterase domain (Figure 1A). The α/β hydrolases consist of a superfamily of enzymes, such as thioesterases, proteases, lipases, peroxidases and epoxide hydrolases (Nardini and Dijkstra, 1999). These hydrolases are highly diverse in sequences, but their secondary structure arrangements and catalytic triad residues are highly conserved, including the catalytic triad consisting of a nucleophile residue (Ser, Cys, or Asp), an acidic residue (Asp or Glu) and a His residue (Nardini and Dijkstra, 1999; Lord et al., 2013). CpTEII contained all three catalytic triad residues including Ser^128^, Asp^278^ and His^309^ (Figure 1B), which corresponded to the triad Ser^94^, Asp^200^, and His^228^ of RifR, a classic type II thioesterase from the rifamycin synthetic pathway in *Amycolatopsis mediterranei* (Claxton et al., 2009). The Ser^128^ and His^309^ residues in CpTEII were in the highly conserved Gx**S**xG motif and the less conserved Gx**H**FL motif, respectively (Figure 1B) (Kotaka et al., 2009; Wang et al., 2014). These observations strongly implied that CpTEII and its orthologs in other *Cryptosporidium* species are type II thioesterases.

### CpTEII Prefers to Hydrolyze Decanoyl-CoA

**Figure 2:**
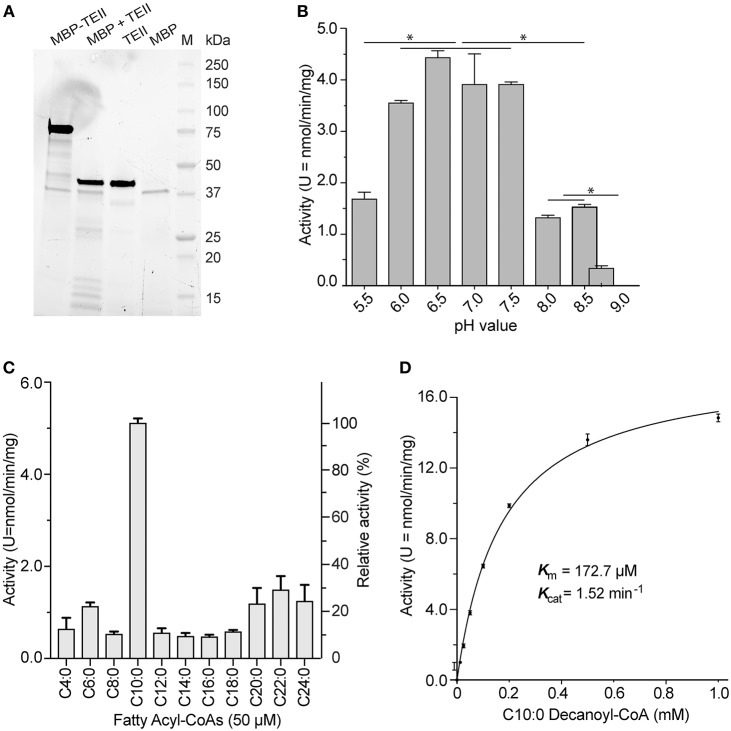
Biochemical features of CpTEII. **(A)** SDS-PAGE analysis of the purified MBP-CpTEII fusion protein, MBP-CpTEII after TEV cleavage, Purified CpTEII and MBP. **(B)** Effect of pH on the activity of CpTEII toward C10:0 decanoyl CoA (50 μM). The Tukey's multiple comparisons test was used to analyze the effect of pH on the CpTEII activity (* indicates *p* value ≤ 0.05). **(C)** Determination of the substrate preference of CpTEII using various even-chain fatty acyl-CoA thioesters. **(D)** Michaelis-Menten kinetics of CpTEII toward decanoyl-CoA. Vertical bars in **(B–D)** represent standard error (SE) derived from at least duplicated measurements.

The recombinant CpTEII protein was successfully expressed and purified as an MBP-fusion protein. The purified fusion proteins, including several smaller sized proteins, could be cleaved into MBP, CpTEII and smaller fragments by TEV protease (Figure 2A). After cleavage, the CpTEII fragment, containing 30 extra amino acids of linker and His-tag, is 43 kDa, which is larger than native protein and MBP (Figure 2A). We first observed that both the uncleaved MBP-CpTEII fusion protein and the CpTEII portion released by TEV cleavage displayed virtually the same enzyme activity (data not shown). We then used the intact MBP-CpTEII fusion protein in all subsequent biochemical experiments. MBP-tag did not show much activity on most tested substrates (Figure S2). To exclude the MBP contribution to the CpTEII activity, MBP-tag only was used as a negative control for background subtraction. The DTNB-based assay was performed at pH 7.4 based on the optimal activity of CpTEII at near neutral pH conditions on its most preferred substrate decanoyl-CoA (Figure 2B). Previous study showed that higher concentration of long chain acyl-CoA substrate inhibits a thioesterase activity, and adding BSA can reverse or decrease the inhibition (Westin et al., 2004). Adding BSA in the reaction improved the CpTEII activity on long chain acyl-CoA, especially on C22:0 behenoyl-CoA, so BSA was included in all the reaction for CpTEII substrate preference assay (Figure S3). More detailed analysis revealed that the hydrolysis activity of CpTEII on C10:0 decanoyl-CoA was ~3 to 10 times higher than on other tested acyl-CoA thioesters. Among other substrates, the activities of CpTEII on very long chain acyl-CoAs (i.e., C20:0, C22:0, and C24:0) were higher than others (Figure 2C). On decanoyl-CoA, CpTEII followed a Michaelis-Menten kinetic (K_m_ = 172.2 μM, K_cat_ = 1.52 min^−1^) (Figure 2D).

### CpTEII Is Differentially Expressed During the Parasite Life Cycle

**Figure 3:**
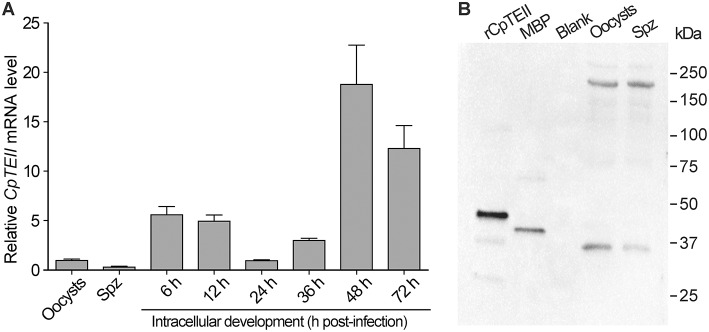
*CpTEII* gene is expressed in all life stages of parasites being tested. **(A)** The levels of *CpTEII* transcripts were tested by qRT-PCR in oocysts, sporozoites, and intracellular parasites infecting host cells for various times. The relative levels were normalized to the *C. parvum* 18S rRNA (*Cp18S*) and showed in relative to the level in oocysts using the empirical $2^{{-\Delta \Delta C}_{T}}$ equation. **(B)** Western blot analysis of purified CpTEII, MBP and crude extracts from *C. parvum* oocysts and free sporozoites (Spz) using affinity-purified rabbit anti-CpTEII polyclonal antibodies.

At RNA level, the *CpTEII* transcripts were detectable in all tested parasite life cycle stages (Figure 3A). There were apparent three levels of *CpTEII* transcripts that were correlated with the parasite cell cycles: (1) the highest levels in the later intracellular developmental stages at 48 and 72 h post-infection (hpi) time points; (2) the moderate levels in the intracellular stages at 6, 12, and 36 hpi; and (3) the lowest levels in the oocysts, free sporozoites, and the intracellular parasite at 24 hpi.

At the protein level, the affinity-purified polyclonal antibodies were able to detect the recombinant CpTEII protein and MBP tag (Figure 3A). The presence of anti-MBP antibodies had no effect on detecting CpTEII in *C. parvum* due to the fact that the parasite possesses no MBP homologs (Zhang et al., 2015). However, western blot detected two major proteins from the crude extracts of the parasite oocyst and sporozoites at ~35 and ~200 kDa, respectively (Figure 3B). The 35 kDa protein was slightly smaller than the predicted 39 kDa of CpTEII. While the nature of the 200 kDa protein remains to be resolved, the antibody specificity could be justified by the facts: (1) presoaking the polyclonal antibody with recombinant CpTEII protein could eliminate or highly decrease the signals from both 35- and 200-kDa bands, as well as signals from purified CpTEII proteins in the blots (Figure S4), and (2) there is no second protein in *C. parvum* sharing a stretch of ≥5 identical amino acids with CpTEII.

**Figure 4:**
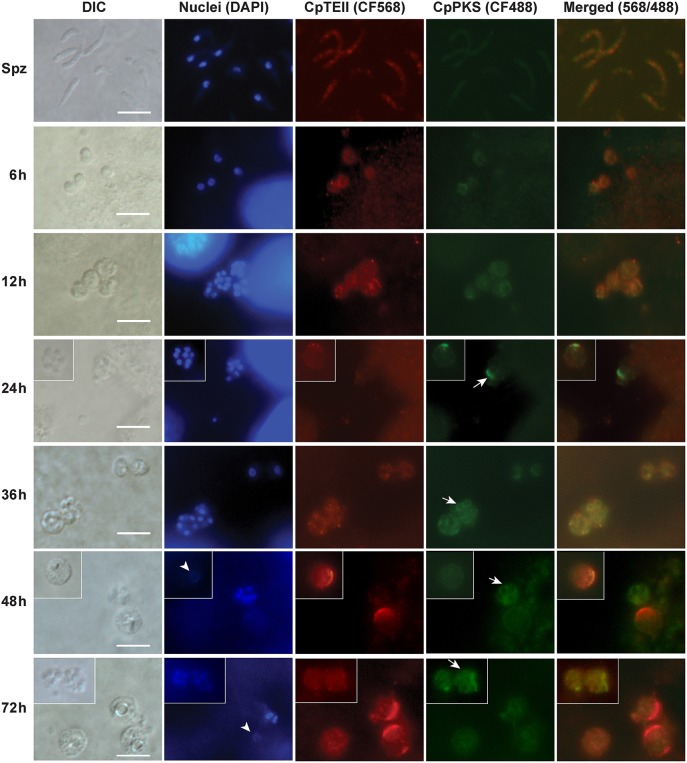
Indirect immunofluorescence microscopic detection of CpTEII and CpPKS protein in different life stages of *C. parvum*. Anti-CpTEII antibodies stain highly on some later stage parasites (48 and 72 h post-infection), which are probably macrogamonts with large or unstainable nuclei by DAPI (arrowhead), and relative weak signal can be observed on sporozoites and other parasites. CpPKS staining signal is very faint on sporozoites and weak in 6 and 12 h postinfection stage parasites, and become stronger on some 24, 36, 48, and 72 h postinfection stage parasites (arrow). DIC, differential interference microscopy; DAPI, 4′,6-diamidino-2-phenylindole for staining nuclei; Merged, superimposed images of CpTEII and CpPKS. Bar = 5 μm.

In agreement with qRT-PCR data, indirect immuno-fluorescence microscopy (IFM) also detected the strongest signals of CpTEII protein in *C. parvum* at 48 and 72 hpi time points, although signals were detectable in other parasite developmental stages (Figure 4). More specifically, brightest fluorescence signals were present only in large parasite cells with no or weak DAPI straining, which is characteristic to the macrogamonts (female gamonts) with large diffuse nuclei (Figure 4) (Wilke et al., 2018).

In IFM experiment, we also co-stained CpTEII with CpPKS1 protein using chicken IgY antibodies (Zhu et al., 2002), because CpPKS is one of the potential targets for CpTEII to edit non-reactive residues or aberrant intermediates and/or to release final products. Here the signals from CpPKS1 were weak in sporozoites and intracellular parasites at 6 and 12 hpi, but became higher in parasite cells at 24–72 hpi (Figure 4). The brightest staining for both CpTEII and CpPKS1 proteins were in later post infection stage parasites with CpTEII especially works on female gamonts, so we speculated that both proteins may play important roles in the parasite sexual development. However, their signals were not fully overlapped, which could be explained by that it is unnecessary for all CpTEII proteins to be distributed in the close vicinity of CpPKS1 and there are other targets of CpTEII such as CpFAS1.

## Discussion

In the present study, we have characterized the molecular and biochemical features of a type II thioesterase (TEII), not previously studied in other protozoans so far. Thioesterases (TEs) are a superfamily of enzymes catalyzing the hydrolysis of various thioesters. In the biosynthesis of fatty acids, polyketides and non-ribosomal peptides, the releasing of the acyl chains from acyl-carrier proteins (ACPs) is commonly catalyzed by two types of TEs. Among them, a type I TE (TEI) is a domain of a multifunctional fatty acid synthase (FAS), polyketide synthase (PKS), or non-ribosomal peptide synthase (NRPS), whereas a type II TE (TEII) is a discrete protein (Gokhale et al., 1999; Kohli et al., 2001; Boddy et al., 2003; Kotowska and Pawlik, 2014).

The function of TEII was first studied with rat type I FAS complexes, in which the purified TEII from lactating rat mammary gland hydrolyzes medium-chain thioesters to release medium chain fatty acids (e.g., C8:0, C10:0, C12:0), rather than a typical final product of the FAS (i.e., C16:0 palmitic acid) (Libertini and Smith, 1978). Since then, a variety of prokaryotic and eukaryotic TEII enzymes have been reported to play diverse roles, including the removal of non-reactive residues or aberrant intermediates, control of starter units, providing key intermediates, and the release of products (Kotowska and Pawlik, 2014).

For example, the TEII (TylO) from tylosin synthase of *Streptomyces fradiae* removes short-chain fatty acyl thioesters produced by aberrant decarboxylation of malonate-derived moiety from ACP, and the disruption of *tylO* gene could reduce the accumulation of polyketide in *S. fradiae* by >85% (Butler et al., 1999; Heathcote et al., 2001). Similarly, the TEII (RifR) from the hybrid PKS/NRPS rifamycin B biosynthetic cluster in *Amycolatopsis mediterranei* prefers to hydrolyzing decarboxylated acyl thioesters over carboxylated extender units, which is aligned with its correcting function in the removal of aberrant or non-reactive residues (Claxton et al., 2009). By removing non-reactive acyl residues from carrier domain to regenerate the activity of PKSs and NRPSs, TEII enzymes save cells a significant effort for synthesis and degradation of these megasynthases. In the case of 6-deoxyerythronolide B synthase (DEBS) system in *Saccharopolyspora erythraea*, a TEII functions to control the starter unit by selectively hydrolyzing acetyl group, but not propionyl and butyryl group, bound to the ACP of the DEBS loading unit (Hu et al., 2003). In some organisms synthesizing polyether ionophore antibiotics (e.g., monensin, anchangmycin, and nigericin), the TEII enzymes are responsible to release the final products from the PKSs lacking terminal TEI domains (Harvey et al., 2006; Liu et al., 2008). Overall, TEIIs play a significant role in maintaining the accuracy and efficiency of many PKSs and NRPSs.

Among the 23 types of thioesterases (TE1–TE23) as classified by the ThYme database (http://www.enzyme.cbirc.iastate.edu), only TE2, TE16 to TE22 contain the α/β hydrolase fold, while most other types contain HotDog fold (Cantu et al., 2010). Among those containing the α/β hydrolase fold, only TE18 enzymes representing TEII use medium-chain acyl-ACPs as preferred substrates. Our molecular analysis of motifs and active sites of CpTEII, as well as its preference on medium-chain acyl-CoA, particularly on the C10:0 decanoyl-CoA, supports the notion that CpTEII is indeed a type II thioesterase.

The *C. parvum* genome encodes two giant multi-module and multi-domain megasynthases, i.e., CpFAS1 and CpPKS1 (Zhu et al., 2002, 2004; Abrahamsen et al., 2004). Both CpFAS1 and CpPKS1 use a C-terminal reductase domain to release final products, rather than using a type I thioesterase domain. The presence of CpTEII in the parasite raises a question on whether the parasite uses CpTEII as an alternative tool to release the final products from CpFAS1 and/or CpPKS1, or as an editor to remove non-reactive residues or aberrant intermediates, or to control starter units as seen in other organisms (Kotowska and Pawlik, 2014). The substrate preference on decanoyl-CoA suggests that CpTEII may play an editing role to remove undesirable substrates of CpFAS1 or CpPKS1.

Our previous studies showed acyl-ACP ligase (AL) of both CpPKS and CpFAS are specific to loading long chain fatty acids to their assembly line (Zhu et al., 2004; Fritzler and Zhu, 2007). Therefore, the CpTEII is beneficial to the parasite, if it removes the unwanted medium chain substrates from these assembly lines. Previous studies also indicted that the final products synthesized by CpFAS1 and CpPKS1 are expected to be considerably large, i.e., ≥22 or ≥30 carbon units, respectively (Zhu et al., 2002, 2004, 2010; Fritzler and Zhu, 2007). It is possible that the CpTEII also participates in the release of final products from the CpFAS due to its moderate activity on the arachidoyl (C20:0), behenoyl (C22:0), and lignoceroyl (C24:0)-CoA thioesters. The CpTEII activity on substrate with more than 24 carbon chain has not been tested, so that its potential to release final products from CpPKS is unknown. In addition, unsaturated and odd carbon chain acyl-CoAs are also potential substrate of the CpTE II.

In summary, we confirmed that the predicted thioesterase in *C. parvum* is functional and is a type II thioesterase. The CpTEII can hydrolyze fatty acyl-CoA with decanoyl-CoA as a preferred substrate, which leads the speculation that it may function to remove undesirable substrate from CpFAS1 or CpPKS1. Due to its activity on long chain acyl-CoA, the CpTEII may also participate in final product releasing from the CpFAS. Its mRNA and protein are both highly expressed in latter post infection stage parasites, with protein highly expressed on female gamonts, suggests the potential that the CpTEII involves in parasite sexual development, which could be an interesting subject for further investigation.

## Author Contributions

FG and GZ designed the experiments and wrote the manuscript. FG conducted majority of the experiments. HZ and RE assisted the experiments.

### Conflict of Interest Statement

The authors declare that the research was conducted in the absence of any commercial or financial relationships that could be construed as a potential conflict of interest.
