# Supplementary material for: Molecular and Biochemical Characterization of a Type II Thioesterase From the Zoonotic Protozoan Parasite Cryptosporidium parvum
Source: Front Cell Infect Microbiol. 2019 Jun 7;9:199. doi: 10.3389/fcimb.2019.00199 (PMC6568194; doi:10.3389/fcimb.2019.00199)

Figure S1. A multiple sequence alignment of CpTEII orthologs from represenative species. The CpTEII orthologs with query cover ≥50% and expect value ≤ e-5 were retrieved from NCBI protein databases and multiple sequence alignment was performed by using T-coffee program. All TEII orthologs from *Cryptosporidium* spp. were retained, while the nearly identical sequences from other species were removed from the dataset containing 32 sequences from apicomplexans, algae and bacteria. This alignment shows TEII sequences from 15 representative species. The two conserved motifs are shaded. The three residues representing catalytic triad are boxed. Asterisks (*) indicate residues conserved among all listed sequences.

```
Apicomplexa
Cryptosporidium parvum (XP_628405)              RLLCIHGAGGSDQIFVKKDLNQENGIELLVLQLPGRAGRSHEACYEDIATLINDFYPVFKSHFLIPWVIV
Cryptosporidium meleagridis (POM82800)          RLLCIHGAGGSDQIFVRKDLNQENGIELLVLQLPGRAGRSQEACYKDITTLINDFYPVFKSHFLTPWVIV
Cryptosporidium ubiquitum (OII74105)            RLLCIHGAGGSDQIFVKKDLNQENGIELLALQLPGRAGRFHETCYKEITELINDFYPVFKNHFAVPWVLI
Cryptosporidium andersnoni (OII75798)           RILCIPGAGSTDQIFVQAKAPEENNIEILAMVLPGRAQRSGEEHYKSIIDVINDFYPVFEEHFIVPWILI
Cryptosporidium muris (XP_002141400)            RILCIPGAGSTDQIFVQAKAPEENNIEILAMVLPGRAQRSGEEHYKSIIDVINDFYPVFEEHFIVPWILI
Eimeria tenella (XP_013228501)                  RVFCFPGAGMEASIWTGRGTSQKEGVAFLALQMPFRGLRRREAHPRTLRAAAAQALDAMRPLICCPYVLV
Algae
Bathycoccus prasinos (XP_007508988)             RVICFANAGNAEDLFTNEGLGKNYKAELLSVQLPGRGSRRNEPWDPKIQTVIREIAPTLANELDVPYVVV
Ostreococcus tauri (XP_003074702)               RVLCFPNAGNQEDMFTSEGTGKSNECEVLAVQYPGRANRKSEPYAFDIADITVPLLPVVGPKLECPFVII
Ahniella affigens (WP_106891066)                RLFCFAYAGGGASAFASLARSLPSSVEILSVQLPGREGRFHESPLTQLPEVMDGVARAVGDVLDKPYLLF
Bacteria
Xenorhabdus sp (WP_099111618)                   RIFCFPYAGGSNSTFVPFARKIKENVEFLVSQPPGKGARFKEKPYDNMQNLVEDIYTNIKPLLDKPYIFL
Nostoc sp (WP_086691638)                        RLFCFPYAGGSSLIFRSWPDSLPTSVEVCTVELPGRGRQMKLQPYNKIEPLVDAIASVIHPYLDIPFAFF
Bacillus patagoniensis (WP_078393735)           RIICIPYAGGSAAIYKSWEKLAGKDVQLYALQLPGRGGRYEEKPYTQMDNLVIDLFNEIRELFDTPYIIF
Herpetosiphon sp (WP_110518018)                 RLFCFHYAGGTAQRAHQWAKQLDQEVELCAIELPGRGKRFMEPAYTNLDQLLNDLIPVIKPLLEKPFVFF
Silvibacterium bohemicum (WP_082125874)         RLFCFPAAGASANSYWSWGAALPPFLEAFGIQLPGRGERFHEEPTRDMQALVAELAQATRPLRDRPFVFL
Nocardia sp (WP_082373925)                      RLLCLPPGGGSAQLYQRWPALLGPEVEVVAVELPGRGARSLQPPLTDMATLVPVLVAEL--ARDLPLVVF
                                                *. *    *                        * .  .        .                 *
```

```
                                                GxSxG
Apicomplexa
Cryptosporidium parvum (XP_628405)              GHSMGGLIGFELLKRIKFEQPNEELNDEEFKQECREWG-INQNVF-KKGIWEEFEKQLRCDFTMFDSFEM
Cryptosporidium meleagridis (POM82800)          GHSMGGLIGFELLKRIKFIFPEWELNDEEFKQECREWG-INENVF-KKGIWEEFEKQLRCDFTMFDSFEM
Cryptosporidium ubiquitum (OII74105)            GHSMGGLIGFELIKRIKFMFPEWELNDEEFKQECREWG-INENVF-KKGIWEEFEKQLRCDFTMFDSFEM
Cryptosporidium andersnoni (OII75798)           SHSMGGLIAFELLKRLKYIFPQWNMNTEEFKEECRRWG-INNEVF-RKGIWEEFEPLLRSDFHLFDSYNL
Cryptosporidium muris (XP_002141400)            SHSMGGLIAFELLKRLKYIFPQWNMNTEEFKEECRRWG-INNEVF-RKGIWEEFEPLLRSDFHLFDSYNL
Eimeria tenella (XP_013228501)                  GYSMGCWLAFEVLQQAKLLLPLWALDTAAFQNQLRQWN-CNEVLF-QKDMWAAYEPLLRADHNMLDEYQP
Algae
Bathycoccus prasinos (XP_007508988)             GHSVGCWLAVQLVDVLKSNEPLWHLSEREFQDECRRWD-VQEVVF--TGIWKIYHPLMRADFCLFDSYEN
Ostreococcus tauri (XP_003074702)               GHSVGSWVAFEFMQLMRSRMPEWLLDEDAFKEECRRWD-VNELVF--GPLWEVYHDLMRADFHLFDKYDY
Ahniella affigens (WP_106891066)                GHSLGALIAFELTRHFAAPLPSFRLPDEEFIQEIANYKGTPASVLENRELMELVLPRLRADARLFDDYEY
Bacteria
Xenorhabdus sp (WP_099111618)                   GHSLGSRVAFELLRKIAAPLPIISLNDKEFAIALKDFGRVNQQILENDELLSLILPAIKADFKISEEYYY
Nostoc sp (WP_086691638)                        GHSMGGLVSFELARLLRKTLPVINLPELAFIEELRQLKGTPPAVLENAELMQLFLPILRADFAVLETYIY
Bacillus patagoniensis (WP_078393735)           GHSMGALIGYELVRVIRKNMPKTKLSTQDLQNVLQDYNGTPNEILENLEMLEMFLPSIRADFELVEEYCY
Herpetosiphon sp (WP_110518018)                 GHSMGALVSYELSRRLQHLRPQIQLPHQQFWDRLMQLNGTPKDILQHPELVELLTPILRADFAVCETFGY
Silvibacterium bohemicum (WP_082125874)         GHSLGTLIAFELARTLRRRLPLARLPDAQFVERLRELGGSGPEILAEQELMELLMPTLRADFAMDETYSY
Nocardia sp (WP_082373925)                      GHSMGGIVGWELCRRLPR-EPRYETTDEGLRELITRSEGLPSATALDPLFLEYFLPMLRADLTLVNSFEP
                                                  *.*  .  .         *                                    ...*  .   .
```

```
                                                                                              (G/A)xH
Apicomplexa
Cryptosporidium parvum (XP_628405)              NLYPLGVDAQLWSASQDSKVTKD-IMNKWKELLTSNSLDTREIDAQHNFLHDPK
Cryptosporidium meleagridis (POM82800)          NLYPLGVEAQLWSASQDNKVTKD-IMNNWKELLTLNSLDIREIDAQHNFLHDPK
Cryptosporidium ubiquitum (OII74105)            NSYPLGVKAQLWSASQDKKVTKN-IMNKWKELLVHDLHEIREIDAAHNFLHDPK
Cryptosporidium andersnoni (OII75798)           LEYPLGVRVQLWSATEDKRATKS-AMYGWIKLVACGDISEHEIQAGHNFLHNLK
Cryptosporidium muris (XP_002141400)            LEYPLGVRVQLWSATEDKRATKS-AMYGWIKLVACGDISEHEIQAGHNFLHNLK
Eimeria tenella (XP_013228501)                  T-DPLDIPCSVFRGSRDLQLADNSLFEGWFRLLRS-GPQVQTLDGDHGLVFDPN
Algae
Bathycoccus prasinos (XP_007508988)             DLKTFTSDVTAFHGTNDRMISKS-MVEKWETMT-TGAFALEIVQGNHLFPLEKE
Ostreococcus tauri (XP_003074702)               AHENFAWPLTMFYGESDRMITRE-MCAFWETHT-TGEFELVNIPGHHLFPLQKD
Ahniella affigens (WP_106891066)                RAGPLPCPIVAFGGTDDPHVNLD-ELAAWRELT-T-TFSSRVFEGDHFFIHGNQ
Bacteria
Xenorhabdus sp (WP_099111618)                   IGESFACPLTVFGGTDDPLVAEH-ELYSWFDLF-EQDKNVILFQGRHFFIDDNH
Nostoc sp (WP_086691638)                        TSPPLECPITVFGGLQDSEVGCD-ELQAWQEQT-KADFNLHMFPGDHFFLHSSQ
Bacillus patagoniensis (WP_078393735)           TQCPLNCPITALWGTKDHLVNIR-EIEKWKYHT-DKKFQIKQFEGDHFFIHNYT
Herpetosiphon sp (WP_110518018)                 NNIVLDCPMVVYGGLDDPSVAAA-ELELWRNHT-SNACEIELMAGDHFYLHNPS
Silvibacterium bohemicum (WP_082125874)         TAPPLACPITAWGGSEDAELSPE-DLALWQHQT-ASSFELQIFQGNHFFVHSQL
Nocardia sp (WP_082373925)                      EPPPLRCPLRVYRGAADPLVGDD-EANAWLAET-QGDARVHTFDGGHFFPRDLP
                                                                *           *                 *
```

Figure S2. MBP-tag itself had no or little effect on the CpTEII activity assay. The assay was performed in 200 µL of HEPES buffer (0.1 M, pH 7.4) containing 50 mM of KCl, 50 µM of a specified fatty acyl-CoA, 50 uM of DTNB and 10 µg of MBP-CpTEII or MBP-tag only.

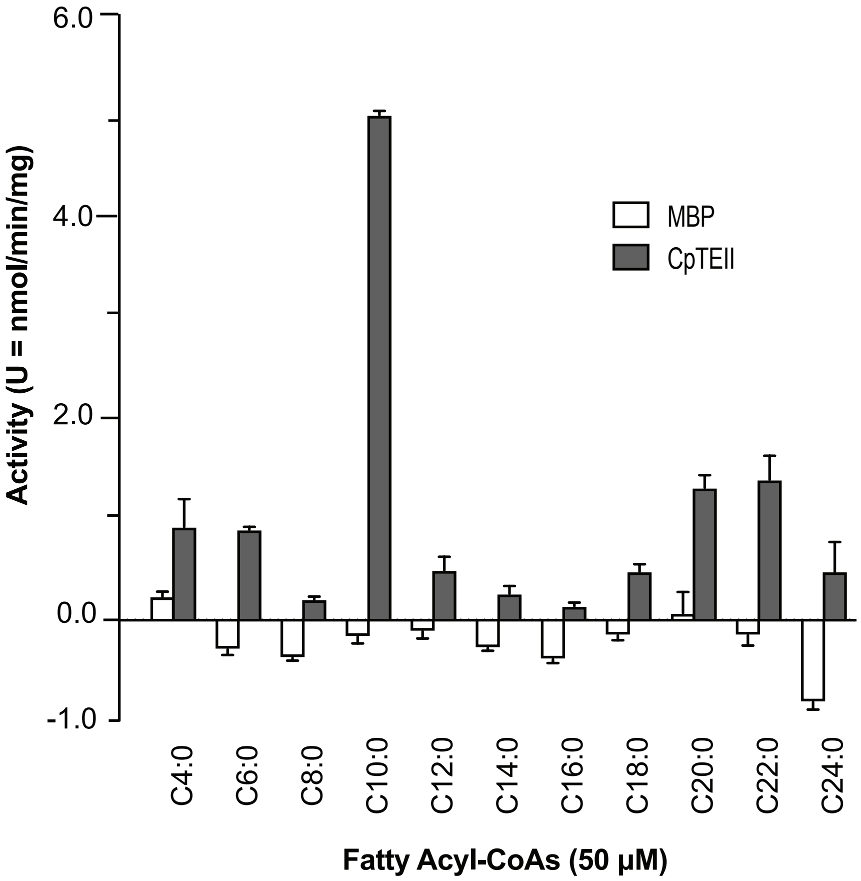

Figure S3. Effect of bovine serum albumin (BSA) on CpTEII activity. Significant improvement of CpTEII activity on behenoyl coenzyme A (C22:0 CoA) was observed. The assay was performed in 200 µL of HEPES buffer (0.1 M, pH 7.4) containing 50 mM of KCl, 50 µM of a specified fatty acyl-CoA and 50 uM of DTNB. The reaction started by adding MBP-CpTEII only or MBP-CpTEII with BSA at molar ration 4.5:1.

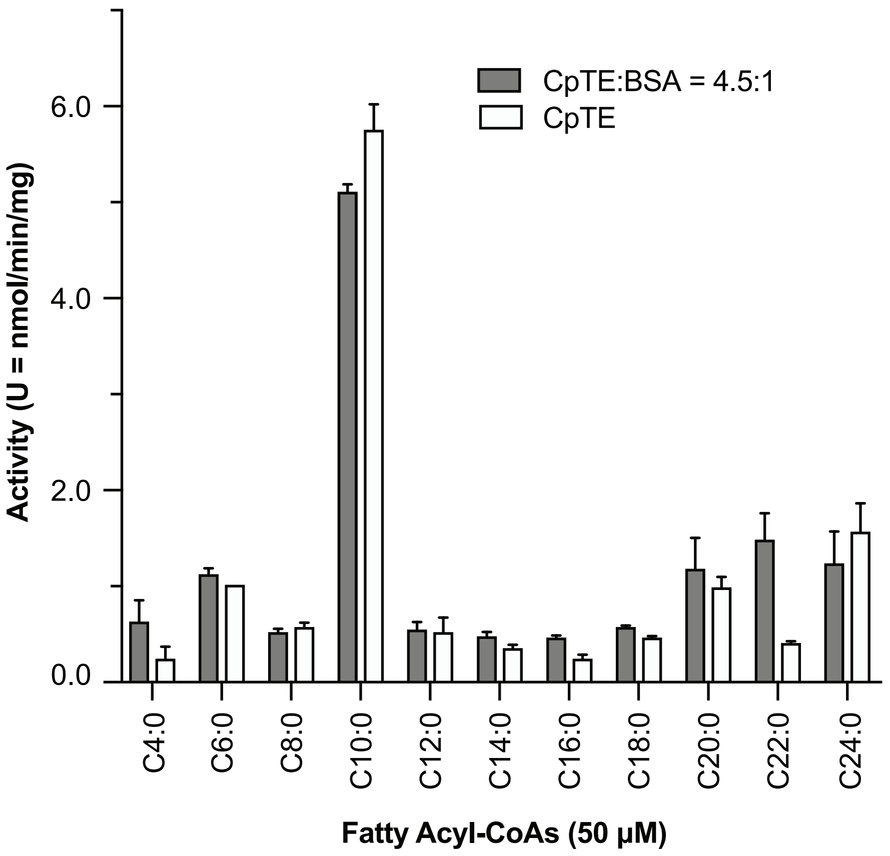

Figure S4. The anti-CpTEII antibodies specificity assay by western blotting. (A) The purified CpTEII (0.1 µg), MBP (0.1 µg), and crude extracts of oocysts (1 x 10^7/lane) and sporozoites (Spz) (~4 x 10^7/lane) were separated by 4-20% SDS-PAGE. (B) The fractionated proteins from the gel (A) were transferred onto nitrocellulose membrane for western blot analysis. The membranes were probed with anti-CpTEII antibody (1:1,000 dilution) (left) or the antibody (1:1,000 dilution) presoaked with MBP and CpTEII proteins immobilized on nitrocellulose membrane, followed by incubation with a goat anti-rabbit secondary antibody conjugated with horseradish peroxidase (HRP; 1:20,000 dilution). The membranes were developed with SuperSignal West Femto maximum sensitivity substrate and imaged using ChemiDoc XRS+ system (Bio-Rad).

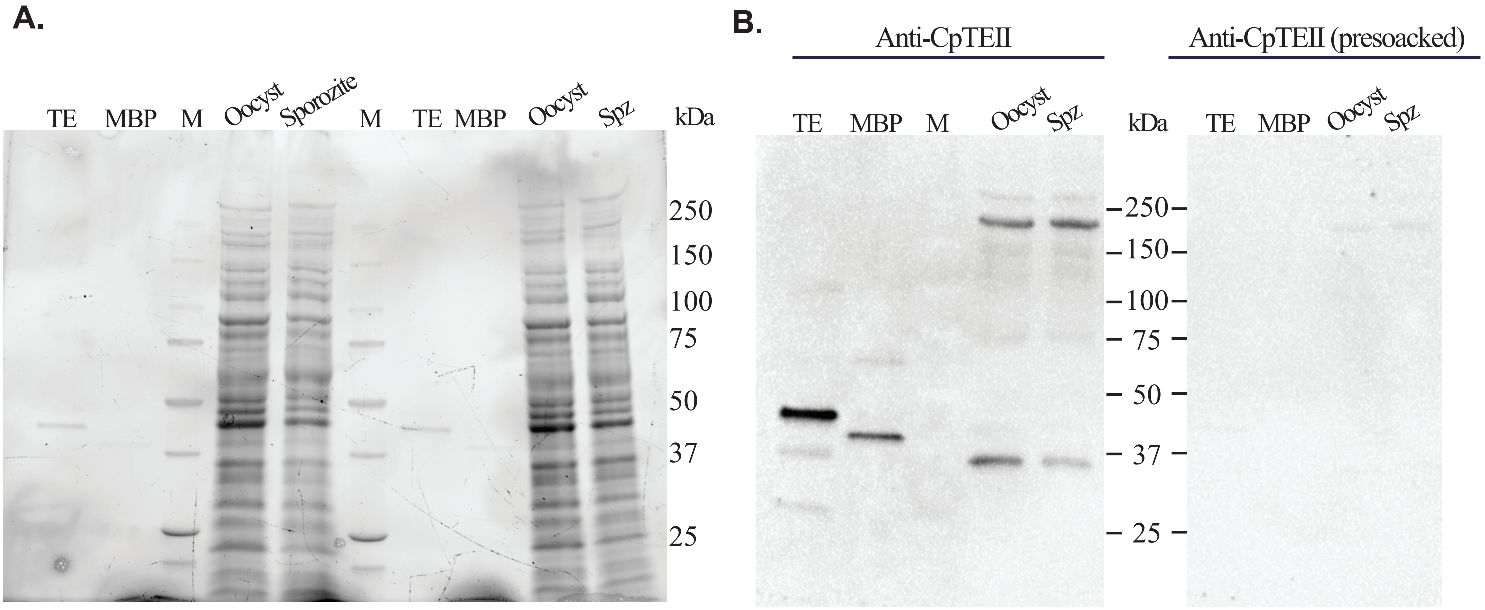

Supplement: Supplementary file 1 [file Presentation_1.pdf]
